# Insulin Resistance Promotes Early Atherosclerosis via Increased Proinflammatory Proteins and Oxidative Stress in Fructose-Fed ApoE-KO Mice

**DOI:** 10.1155/2012/941304

**Published:** 2012-03-07

**Authors:** Beatriz Cannizzo, Agustín Luján, Natalia Estrella, Carina Lembo, Montserrat Cruzado, Claudia Castro

**Affiliations:** ^1^Laboratory of Vascular Biology, Institute of Medicine and Experimental Biology of Cuyo (IMBECU)-CONICET, Faculty of Medical Sciences, National University of Cuyo, 5500 Mendoza, Argentina; ^2^Laboratory of Cardiovascular Pathophysiology, Institute of Medicine and Experimental Biology of Cuyo (IMBECU)-CONICET, Faculty of Medical Sciences, National University of Cuyo, 5500 Mendoza, Argentina

## Abstract

High fructose intake induces an insulin resistance state associated with metabolic syndrome (MS). The effect of vascular inflammation in this model is not completely addressed. The aim of this study was to evaluate vascular remodeling, inflammatory and oxidative stress markers, and atheroma development in high-fructose diet-induced insulin resistance of ApoE-deficient mice (ApoE-KO). Mice were fed with either a normal chow or a 10% w/v fructose (HF) in drinking water over a period of 8 weeks. Thereafter, plasma metabolic parameters, vascular remodeling, atheroma lesion size, inflammatory markers, and NAD(P)H oxidase activity in the arteries were determined. HF diet induced a marked increase in plasma glucose, insulin, and triglycerides in ApoE-KO mice, provoked vascular remodeling, enhanced expression of vascular cell-adhesion molecule-1 (VCAM-1) and matrix metalloprotease 9 (MMP-9) and enlarged atherosclerotic lesion in aortic and carotid arteries. NAD(P)H oxidase activity was enhanced by fructose intake, and this effect was attenuated by tempol, a superoxide dismutase mimetic, and losartan, an Angiotensin II receptor antagonist. Our study results show that high-fructose-induced insulin resistance promotes a proinflammatory and prooxidant state which accelerates atherosclerotic plaque formation in ApoE-KO mice.

## 1. Introduction

Insulin-resistant states, including the metabolic syndrome (MS) and type 2 diabetes, have been strongly associated with subclinical and clinical cardiovascular disease (CVD) [1]. Elevated blood glucose, hyperinsulinemia, dyslipidemia and, oxidative stress are central components of MS which are additionally associated with a “proatherogenic” phenotype [2]. There are evidences indicating that structural and functional changes in the vascular wall are involved in cardiovascular alterations associated with MS [3], but the mechanisms underlying are not completely addressed. Previous studies from our group showed that chronic fructose-fed rats exhibited dyslipidemia, hyperglycemia, and endothelial dysfunction, and it was suggested an important role for the renin-angiotensin system (RAS) in the pathogenic mechanisms involved in this model [4].

Arterial remodeling occurs under an atherosclerotic plaque formation [5]. The propensity to accelerated lesion formation in MS may involve altered vascular structure and increased vascular inflammatory response. Delbosc et al. [6] reported that mesenteric arterial media/lumen ratio was higher in fructose-fed rats, and a potential association of soluble adhesion molecules with atherosclerosis has been postulated [7]. Vascular cell adhesion molecule-1 (VCAM-1) is a cytokine-inducible member of the immunoglobulin gene superfamily that is expressed by endothelial cells in regions predisposed to atherosclerosis and at the borders of atherosclerotic plaques [7, 8]. VCAM-1 functions in combination with other adhesion molecules during chronic inflammation, activating NAD(P)H oxidase and endothelial MMPs [9]. Oxidative stress and associated vascular damage are mediators of vascular injury and inflammation in many CVD including atherosclerosis [10]. NAD(P)H oxidase is the major source of vascular reactive oxygen species (ROS) and is expressed in endothelial cells, vascular smooth muscle cells (VSMCs), fibroblasts, and monocyte/macrophages [11]. Extent data strongly support the hypothesis that oxidative stress, induced via activation of NAD(P)H oxidase, plays a causative role in atherosclerosis [12, 13]. ROS are able to regulate cellular growth (hyperplastic or hypertrophic), endothelial dysfunction, cell migration, and inflammation [14]. ROS also induce the expression of matrix-degrading enzymes such as matrix metalloproteinases (MMPs) which are involved in vascular remodeling and are postulated to participate in the pathogenesis of atherosclerosis [15]. We aimed to study the association of fructose intake-induced insulin resistance with the development of atherosclerotic plaque in ApoE-KO mice, an experimental model of cardiovascular complications related to MS, and the relationship between metabolic parameters, vascular inflammation, and oxidative stress.

## 2. Methods

### 2.1. Animals and Diets

All animals were cared in accordance with the *Guiding Principles in the Care and Use of Animals* of the US National Institutes of Health. All procedures were approved by the Animal Research Committee of the Universidad Nacional de Cuyo (protocol approval no. 10089 CICUAL/2009). Male C57/BL6J wild type and ApoE-KO mice 8 weeks of age (20 to 22 g; The Jackson Laboratories, Bar Harbor, ME) were used for this study. The animals were maintained in a 22°C room with a 12 hour light/dark cycle and received drinking water *ad libitum* and were fed a standard commercial chow diet (GEPSA, Argentina). During 8 weeks, animals from each genotype were randomly divided into two groups: control mice (*n* = 10), with free access to tap water; fructose-fed (HF) mice (*n* = 10) receiving 10% (w/v) fructose (Parafarm, Argentina) in their drinking water. Additional group of age-matched ApoE-KO mice were given a control diet or HF during 4 weeks and then were randomized to no treatment, Tempol (Sigma Aldrich, St. Louis, MO, USA; 1 mg/kg of body weight per day), or losartan (Roemmers, Argentina, 10 mg/kg of body weight per day), during 4 more weeks.

### 2.2. Biochemical Determinations

After overnight fasting blood samples for glucose, insulin, triglycerides, and cholesterol determinations were taken from mice, collected from cardiac puncture under anesthesia at the end of the experimental period. The plasma glucose, cholesterol, and triglyceride concentrations were determined using commercial kits by enzymatic colorimetric methods (GT Lab, BuenosAires, Argentina). Insulin was measured by ELISA (Crystal Chem, USA).

### 2.3. Histomorphometric Studies

Mice were euthanized by cervical dislocation and were perfused *in situ* with chilled phosphate-buffered saline. The proximal aorta and the mesenteric artery tree were fixed with 2% paraformaldehyde, paraffin embedded, and mounted in a Micron microtome. Consecutive sections (5 *μ*m thickness) were taken from 2 to 3 regions of the aortic arch separated by *∼*60 *μ*m and from the mesenteric vascular tree. Three cross-sections from each region were stained with hematoxylin/eosin or with Masson's trichrome solution and examined the diameters of the wall and the lumen, displayed in 20X optical microscope and digitized images with the software Image Pro. The linear relationship of superficial lumen/media was calculated for each vessel with the Scion Image software.

### 2.4. Quantification of Atherosclerotic Plaques

Plaque area was quantified by Oil-red-O staining of lipid deposits. Aortas and whole right carotid arteries were incubated for 45 min with Oil red O (0.5% in 60% isopropyl alcohol). Excess stain was removed with 60% isopropyl alcohol. Quantification of atherosclerosis was performed in the aortic arch region up to the descending abdominal aorta and on the bifurcation of the right common carotid arteries by computer-assisted image analysis as previously described in detail [16]. Subsequently images of en face preparations of the whole mounted aorta and the carotid arteries were taken and the percentage of plaques in relation to the entire aortic surface was calculated as plaque score in percent of total area using ImageJ 1.37 v software (NIH).

In another group of animals the whole aorta and right carotid arteries were dissected from mice, perfusion fixed in situ with 2% paraformaldehyde, paraffin embedded, and mounted in a microtome. Atherosclerotic lesion size in cross-sections of the aortic sinus was quantified as the area occupied by hematoxylin/eosin staining.

### 2.5. Determination of Vascular Markers of Inflammation

Paraformaldehyde-fixed sections of aorta or mesenteric arteries were incubated with murine monoclonal antibody against VCAM-1(1 : 500; BD) or rabbit polyclonal antibody against MMP-9 (1 : 200; Santa Cruz), overnight at 4°C, followed by 1 h incubation with FITC or Alexa red conjugated secondary antibody, respectively. Sections were mounted and visualized using fluorescence microscopy. Total area of antigen-specific fluorescence staining per section was quantified using WCIF Image  J imaging software. Positively stained areas were selected and measured using program-specific tools. Two replicate measurements were averaged for each tissue type per mouse (*n* = 4–6) to obtain data for statistical analysis. MMP-9 expression was also determined by Western Blot analysis. Aortic tissues were homogenized in 100 *μ*L lysis buffer supplemented with protease inhibitors (Complete Mini; 1 tablet/1.5 mL; Roche Diagnostics, Mannheim, Germany). Samples were run on 12.5% Tris-HCl gels with Tris/glycine/SDS buffer and the proteins detected; after transfer to PDVF membranes, using rabbit-anti MMP-9 antibody (1 : 500; Santa Cruz Biotechnology, Santa Cruz, CA) and HRP-conjugated secondary antibody (1 : 10.000 Jackson laboratory, US) for 1 h, proteins were visualized by performing luminal-enhanced chemiluminescence. Loading of equal amounts of proteins was confirmed by reproving the membrane with an antibody against tubulin antibody (1 : 100; Santa Cruz Biotechnology, Santa Cruz, CA) and secondary HRP-conjugated antibody (1 : 10.000 Jackson laboratory, US).

### 2.6. Detection of Tissue NAD(P)H Oxidase Activity

The NAD(P)H-driven superoxide production, an estimate of NAD(P)H oxidase activity, was measured in freshly dissected aorta using lucigenin-enhanced chemiluminescence. Longitudinally cut aortas were transferred to 96-well optiplates (Packard, USA) containing 50 mM phosphate buffer, 150 mM sucrose, 100 *μ*M NAD(P)H, and 5 *μ*M lucigenin (N,N′-dimethyl-9-9′-biacridianium, Sigma Aldrich, St. Louis, MO, USA). Luminescence generated by the reaction of tissue-derived superoxide and lucigenin was counted at 20°C with a microplate scintillation counter (Fluoroskan Ascent, Thermo) running in kinetic mode with a 1 min interval for each sample. Luminescence measurements were obtained every 15 s for 25 minutes. Basal chemiluminescence was measured in the tissue placed in the 96-well optiplate (dark-adapted) with phosphate-sucrose buffer and 5 *μ*M lucigenin before 100  *μ*M NAD(P)H addition and subtracted from NAD(P)H-stimulated luminescence. Dried tissues were weighed for calculation of normalized superoxide production expressed as counts per minute and per milligram of dry tissue.

### 2.7. Statistical Analysis

Results are reported as mean ± S.E.M. Significance was determined by nonparametric analysis using Mann-Whitney *U* test to detect a statistical difference between group of interest and its control. Analyses involving more than two groups were done by ANOVA and Bonferroni's post hoc test using Prism-4 software. Differences were considered significant at *P* < 0.05. 

## 3. Results

### 3.1. Effects of High Fructose Diet on Biochemical Parameters

Fructose intake slightly increased cholesterol levels in WT mice compared to WT in a control diet, without changing any of the others variables measured. In ApoE-KO mice 8 weeks of 10% fructose feeding in drinking water significantly increased the concentration of plasma glucose, insulin, and triglycerides, compared to ApoE-KO mice in control diet, with no changes in cholesterol levels (Table 1). 

### 3.2. Effects of High Fructose Diet on Vascular Remodeling

**Figure 1:**
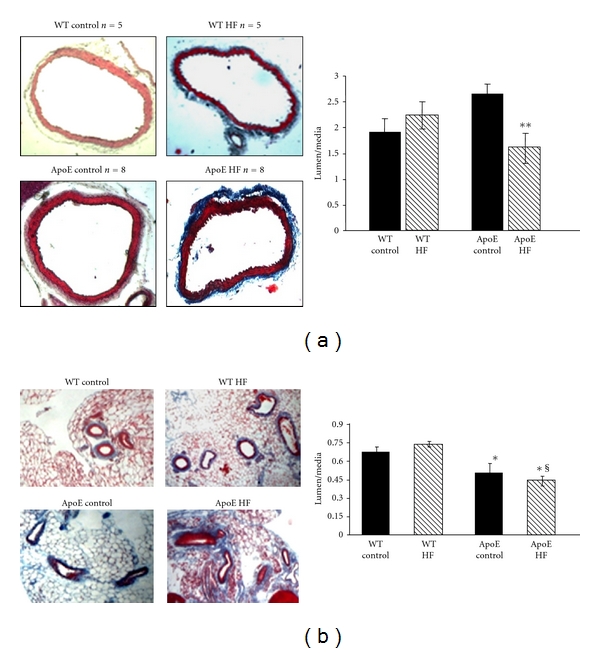
Morphometric analysis of the middle layer of aorta (a) and mesenteric arteries (b) from WT and ApoE-KO mice fed regular chow (Control diet) or high fructose diet (HF). ApoE-KO mice display a decreased lumen/media ratio in vessels walls from fructose-fed ApoE-KO mice. Results are expressed as means (*n* = 8) ± S.E.M **P < 0.001 versus ApoE control in aortic rings; *P < 0.05 versus WT, ^§^ *P* < 0.05 versus ApoE control in mesenteric arteries rings.

We determined the structural changes in the arterial wall by histological analysis of aorta and the mesenteric arteries. In ApoE-KO mice fed with fructose, thickness of the middle layer of both aorta (Figure 1(a)) and mesenteric arteries (Figure 1(b)) significantly increased compared to ApoE-KO mice in a control diet, producing a lower ratio of lumen/media in the vessels of fructose-fed ApoE-KO mice.

### 3.3. High Fructose Diet Enhances Atherosclerosis

**Figure 2:**
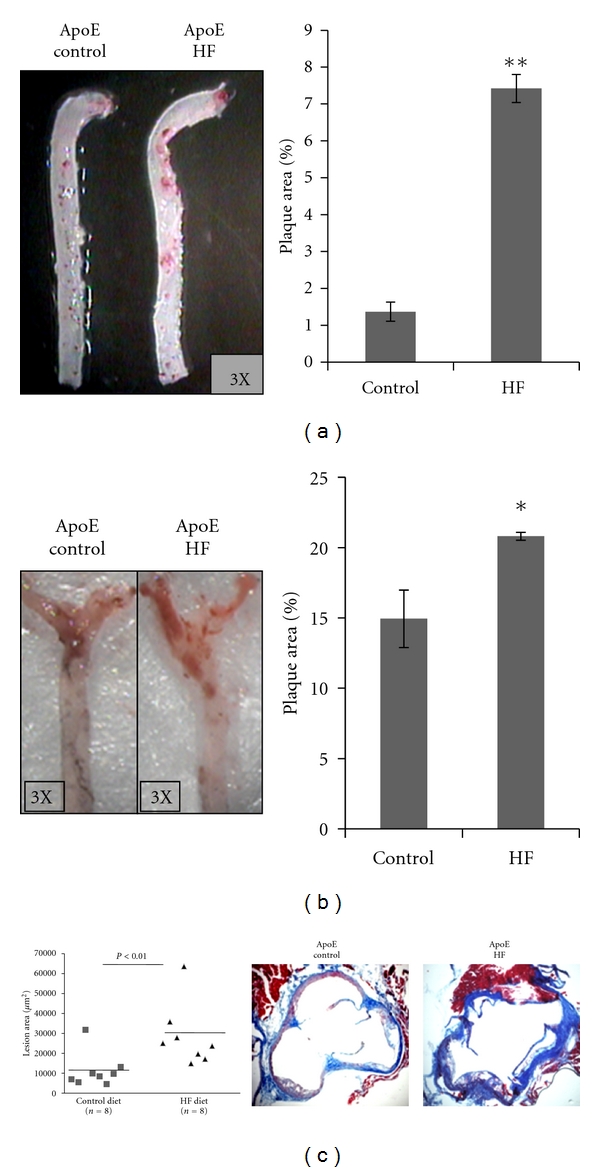
High fructose intake enhances atheroma development. (a) Representative of whole aorta artery and (b) representative of right carotid artery staining with Oil-red O. Extent of atherosclerosis quantified as percent of plaque area in ApoE-KO mice fed control diet and HF diet. (c) Cross-sections from the aortic sinus were stained with hematoxylin and eosin to quantify the lesion area. Results are expressed as means (*n* = 8) ± S.E.M *P < 0.05; **P < 0.01 versus control diet.

We determined the effects of fructose feeding on the development of atherosclerosis in arteries from ApoE-KO mice. We quantified the area of atheroma plaque by computerized morphometry using two independent approaches: (1)* en face* Oil red O-stained mouse aortas and right carotid arteries and (2) quantification of the lesion area in aortic sinus cross-sections. Fructose-treated mice displayed a significant induction in the area of Oil Red O-stained atherosclerotic plaques in both aorta and right carotid arteries (Figures 2(a) and 2(b), resp.), as compared with untreated mice. HF diet increased the lesion area in the aortic sinus in ApoE-KO mice (Figure 2(c)).

### 3.4. Effect of Fructose on Vascular Inflammation

**Figure 3:**
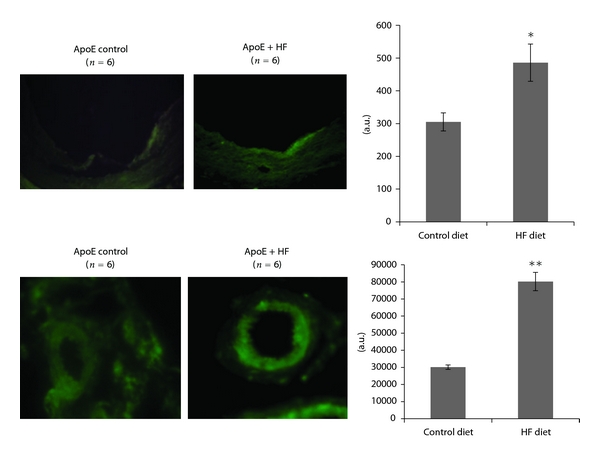
Immunofluorescence detection of VCAM-1 expression (green) in aorta and mesenteric arteries of ApoE KO mice fed control diet or fructose-rich diet (HF). Quantification of the fluorescent signal was performed by ImageJ software. Results are expressed as means ± S.E.M *P < 0.05; **P < 0.01 versus control diet.

Vascular adhesion molecule (VCAM-1) is a hallmark of vascular inflammation. Immunofluorescence detection revealed a markedly increased VCAM-1 expression in aorta, especially on the endothelium, and within the vascular wall in mesenteric arteries from ApoE-KO mice fed with HF diet (Figure 3). 

**Figure 4:**
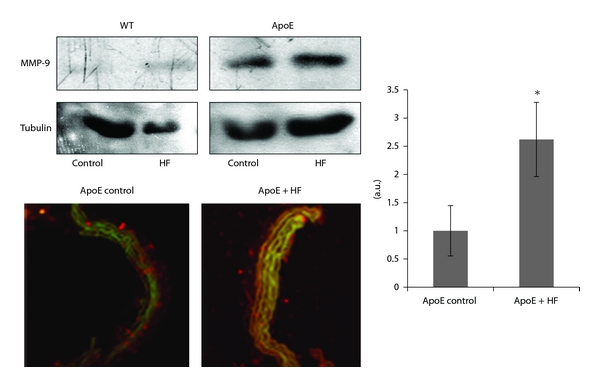
Expression of MMP-9 in aorta from ApoE KO and WT mice fed control diet or fructose-rich diet (HF) determined by Western Blot and immunoflorescence detection. Quantification of the fluorescent signal was performed by ImageJ software. Results are expressed as means ± S.E.M *P < 0.05 versus control diet. (MMP-9: red; Actin: green).

We also evaluated the expression of metalloproteinase 9 (MMP-9), another vascular inflammatory marker involved in remodeling. MMP-9 expression, examined by immunohistochemistry and western blot, clearly increased in fructose-fed ApoE-KO aorta tissue (Figure 4).

### 3.5. Effect of Fructose on NAD(P)H Oxidase Activity

**Figure 5:**
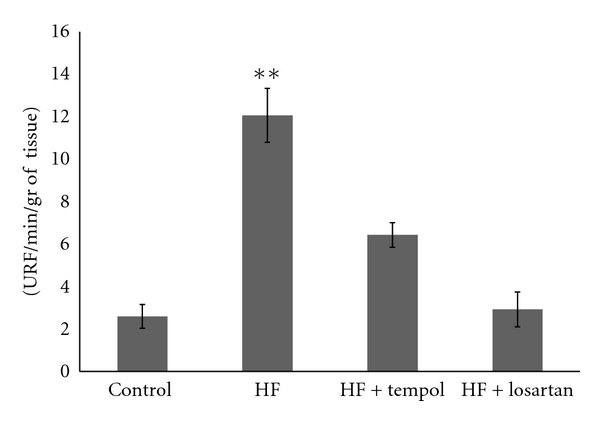
NAD(P)H oxidase activity (relative light units: RLU/mg dry tissue) in aorta of mice fed with control diet (*n* = 8) and mice fed with HF diet (*n* = 8) alone or with Tempol (*n* = 8) or losartan (*n* = 8). Results are expressed as means ± S.E.M. *P* < 0.001  versus control.

NAD(P)H oxidase contributes to basal vascular superoxide production. HF diet increased significantly the NAD(P)H oxidase activity, suggesting the involvement of oxidative stress in this model. Four-week treatment with Tempol, a membrane permeable superoxide dismutase mimetic, or with losartan, an Angiotensin-II receptor 1 (AT1) antagonist, significantly reduced vascular NAD(P)H oxidase activity in aortas from ApoE KO mice in a HF diet (Figure 5).

## 4. Discussion

Most of the studies that utilize fructose intake to develop an experimental model of MS use rat, an animal model that is resistant to develop atheroma plaques and is not suitable for studying inflammatory pathways implicated in the development of atherosclerosis [17]. In this study we induce a MS, characterized by hyperglycemia, hypertriglyceridemia, and hyperinsulinemia in ApoE-deficient mice, an atherogenic-prone animal model, to investigate the effect of insulin resistance induced by fructose feeding on vascular remodeling, vascular inflammation, oxidative stress, and atheroma development. We first noticed that high-fructose diet promoted vascular remodeling, which is involved in the pathogenesis of atherosclerosis. Due to the hemodynamic, histological, and biological particularities in large and small vessels, it is of great interest to investigate structural changes in our model. Turnover of extracellular matrix components and cell migration causes structural changes in the vessel wall with either outward (adventitial/media) or inward (lumenal) protrusion. It is postulated that outgrowth remodeling occurred also in mesenteric artery [18], so we measured the medial thickness of both large and small arteries. We demonstrated that in fructose-fed ApoE-KO mice, the media cross-section area from aortas and mesenteric arteries were significantly increased while lumen diameter was normal. This remodeling format is similar to that found in fructose-fed rats by Puyo et al. [19].

One important feature we found was that fructose-rich diet promotes a proatherogenic state in ApoE-KO mice, independently of hypercholesterolemia, leading to accelerate aortic and carotid atheroma development. It has been demonstrated the involvement of inflammation in vascular remodeling and atherogenesis, with the expression of VCAM-1 in the vascular wall [17, 20]. Here we observed that intimal VCAM-1 expression in small and large caliber arteries was significantly increased in fructose-treated ApoE KO mice compared with control chow mice. 

Different components of the MS have been identified as possible stimulus for the synthesis and activity of MMPs. Diverse MMPs have been identified in atherosclerotic plaques and in regions of foam cell accumulation and have been directly associated with plaque remodeling [21] as well as plaque vulnerability [22]. In our study we found that high fructose diet enhanced expression of MMP-9, a gelatinase enzyme, in atheroma-free area of the vessels during early stages of atherosclerosis development.

Oxidative stress and inflammation processes are key components of atherosclerosis, from fatty streak formation to plaque rupture and thrombosis [23, 24]. Diets rich in fructose can alter cellular metabolism via several pathways, thereby accelerating oxidative stress. Fructose feeding results in the activation of the renin-angiotensin system [25], and it is well established that angiotensin II is associated with oxidative stress [26]. This oxidative stress is characterized by overproduction of ROS and is dependent on the activation of NAD(P)H oxidase. We found that fructose-rich diet increased NAD(P)H oxidase activity in ApoE-KO mice, and this enhancement is diminished by Tempol, a superoxide dismutase mimetic, and by losartan, an Angiotensin-II receptor 1 (AT1) inhibitor, corroborating the role of superoxide generation in fructose-induced insulin resistance and suggesting the involvement of the renin-angiotensin system in the enhanced oxidative stress in our model.

Our results show that insulin resistance associated with MS promotes the initial stages of a proinflammatory and a prooxidant state. These changes potentially contribute to enhancing vascular remodeling and atheroma plaque development and placing the insulin resistance as a potent atherogenic risk factor.

## Figures and Tables

**Table 1:** Average plasma glucose, insulin, and lipid levels in WT and ApoE-KO mice fed with control diet or high fructose diet.

	Glucose (mg/dL)	Insulin(ng/mL)	Cholesterol (mg/dL)	Triglycerides (mg/dL)
WT-control diet	161.2 ± 0.6	nd	71.41 ± 1.5	91 ± 1.2
WT-fructose fed	142.7 ± 5.4	nd	130.35 ± 6.3^*♯*^	83.8 ± 0.7
ApoE-control diet	143.4 ± 25.9	0.85 ± 0.03	391.6 ± 6.0	114.10 ± 3.5
ApoE-fructose fed	216.2 ± 22.2*	1.27 ± 0.19*	382.0 ± 34.5	190.2 ± 18.2*

Values are the mean ± SD (*n* = 12)^*♯*^ *P* < 0.05 versus WT control diet, *P < 0.001  versus ApoE control diet.

## Abbreviations

ApoE-KO: ApoE-deficient mice
NAD(P)H: Nicotinamide adenine dinucleotide phosphate
ROS: Reactive oxygen species
VCAM-1: Vascular cell adhesion molecule-1
MMPs: Matrix metalloproteases.
